# LFC study: Protocol for a longitudinal follow-up cohort study on ageing and mental health in community-dwelling older adults in Singapore

**DOI:** 10.1016/j.mex.2024.102606

**Published:** 2024-02-06

**Authors:** Zhi Hao Lim, Ted Kheng Siang Ng, Zhiming Bao, Junhong Yu, Rathi Mahendran

**Affiliations:** a Department of Psychological Medicine, Yong Loo Lin School of Medicine, National University of Singapore, 12 Science Drive 2, MD1 – Tahir Foundation Building, 117549, Singapore; b Department of Internal Medicine, Rush Institute for Healthy Aging, Rush University Medical Center, Chicago, IL, USA; c Department of English Language and Literature, Faculty of Arts and Social Sciences, National University of Singapore, The Shaw Foundation Building, Block AS7, Level 5, 5 Arts Link, Singapore; d Psychology, School of Social Sciences, Nanyang Technological University, 50 Nanyang Ave, 639798, Singapore; e Mind Science Centre, National University of Singapore, Mind Care Clinic @ SBF, 160 Robinson Road, #05-07 SBF Center, 068914, Singapore

**Keywords:** Cognitive function, Older adults, Depression and anxiety symptoms, Mental Health, LFC Study: protocol for a longitudinal follow-up cohort study on ageing and mental health in community-dwelling older adults in Singapore

## Abstract

The rapid pace of population ageing worldwide has prompted the need to better understand the ageing process. The current study, titled the Longitudinal Follow-up of the CHI (LFC) study, was a 3-year follow-up study of an earlier study titled the Community Health and Intergenerational (CHI) study. The LFC study looked to examine longitudinal changes in their cognitive functioning and psychosocial outcomes across the 3-year period. Additionally, the current study built upon the earlier CHI study by collecting neuroimaging data and exploring the long-term effects of non-pharmacological interventions, which were not examined in the prior study. A total of 653 community-dwelling participants from the baseline CHI study cohort were invited to take part in the LFC study, where they underwent a battery of neuropsychological assessments, psychosocial questionnaires, a Magnetic Resonance Imaging scan and a voice recording segment. The current study would holistically track longitudinal changes in cognitive functioning and psychosocial outcomes in the ageing population in Singapore. Unique associations between linguistics and neuroimaging data alongside cognitive and psychosocial outcomes would be explored. This study also serves to guide the development of new interventions for older adults and assist in improving the well-being of the local and global ageing population.

## Graphical abstract

**Figure:**
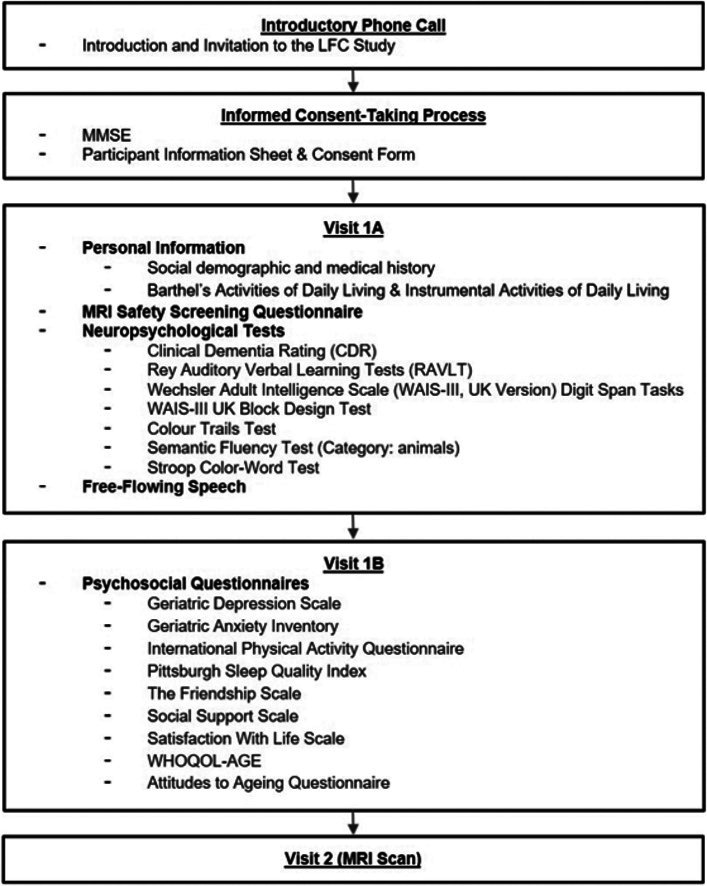

Specifications table

**Table:** 

Subject area:	Neuroscience
More specific subject area:	Longitudinal changes in cognitive functioning and mental health within older adults
Name of your protocol:	LFC Study: protocol for a longitudinal follow-up cohort study on ageing and mental health in community-dwelling older adults in Singapore
Reagents/tools:	Not applicable
Experimental design:	Prospective cohort design, which allows for the longitudinal investigation of relationships between cognitive function, psychosocial outcomes as well as other multidisciplinary outcomes that were measured in the earlier CHI study cohort
Trial registration:	Not applicable
Ethics:	This study had obtained ethics approval from the National University of Singapore Institutional Review Board (NUS IRB-Reference Code: NUS-IRB-2021–162). Written informed consent was obtained from all potential participants before enrolment.
Value of the Protocol:	• The investigation of the effects of non-pharmacological interventions, as well as associations between cognitive decline, psychosocial outcomes, neuroimaging and speech data would contribute greatly to relevant literature and assist with the understanding of the ageing process. • The current study assessed cognitive functioning through a two-step process, where clinical dementia rating and neurocognitive assessments were administered before research diagnoses of normal ageing, mild cognitive impairment or dementia were made during consensus meetings with expert clinicians. This had been previously identified as one of the best available ways to identify those with a target condition amongst a participant pool and represents a robust and comprehensive way to assess cognitive status. • The joint investigation of neuroimaging, linguistics and psychosocial measures in the context of age-related cognitive impairment could be vital in informing the development of interventions to delay cognitive decline in the local and global context.

## Introduction

Population ageing has seen substantial growth in the 21*st* century, with many countries seeing a shift in population distribution towards older ages [1]. The World Health Organization (WHO) projects that the population of individuals aged 60 years and above will double and reach 2.1 billion by 2050 [1]. In Singapore, the proportion of people aged 60 and above is predicted to rise from 13.3 % in 2010 to 31.9 % in 2050 [2]. Accelerated population ageing represents a global challenge, as ageing has been widely associated with cognitive impairment [3]. For example, a meta-analysis revealed that the prevalence of dementia doubled with every 6.7 years increment in age in South-East Asia [4]. In turn, age-related cognitive impairment has been linked with a multitude of risk factors and health concerns, including psychosocial variables, speech and neurological deterioration. Specifically, psychosocial variables pertain to an individual's psychological and social state which in this case can positively or negatively influence their cognitive health. For instance, depression and anxiety have been associated with cognitive deficits in older adults [5,6]. Cognitive impairment has also been linked to linguistic features including pauses as well as slower speech and articulation rates [7,8]. Given the wide range of health issues associated with ageing, the rapid pace of population ageing prompts a pressing need to better understand these factors.

The Community Health and Intergenerational (CHI) baseline study (*n* = 993) was started in 2018 to study these age-related issues in the local context [9]. Participants of the CHI study consisted of community-dwelling older adults aged 60 to 91 from the Toh Yi estate in Singapore and other areas in a 10 km radius from the research site at Hannah Active Ageing Centre (HAAC). They underwent neuropsychological assessments, interviews, blood and dental plaque collection as well as cardiovascular examinations as per study procedures. These provided comprehensive assessments of the health profiles of participants as well as their baseline cognitive functioning and psychosocial outcomes. Data collection for the CHI Study began on 1*st* February 2018 and ended on 26*th* October 2021. Additionally, the CHI study acted as a recruitment platform for other randomized trials that investigate non-pharmacological interventions (NPIs). For example, studies conducted by Shefaly et al. [10] and Low et al. [11] investigated the effects of respective NPIs, including face-to-face mindfulness training and dietary counselling, on the well-being of older adults from the CHI study cohort. Participants who received the face-to-face mindfulness intervention expressed an improvement in their overall well-being and quality of life. Similarly, participants in the dietary counselling intervention group showed a significant increase in dietary quality and decrease in serum low density lipoprotein cholesterol level. Taken together, current evidence has shown that NPIs from the CHI study can be effective in improving the well-being of older adults.

Presently, we initiated a follow up to the CHI study — the Longitudinal Follow-up of the CHI (LFC) study. This was a 3-year follow-up of the CHI study cohort to examine longitudinal changes in their cognitive functioning and psychosocial outcomes, including depression and anxiety symptoms, sleep quality, life satisfaction, quality of life, attitudes towards ageing, social connectedness and perceived social support. Although such associations had been presented in studies examining Western populations, this inquiry remains understudied in the Asian population. Hence, it would be crucial to investigate how psychosocial variables change with time in this specific Asian cultural context, as this would contribute to existing literature regarding the course of ageing and its associated mental health concerns in the Singapore population.

The LFC study also looked into the longitudinal associations of cognitive functioning and psychosocial outcomes with linguistics and neuroimaging measures. Significantly, the latter measure was not collected in the CHI study and thus the LFC study hoped to advance this area of research. The investigation of multimodal neuroimaging data would serve multiple objectives and allow us to test several hypotheses. First, it would enable us to develop brain-based prediction models so as to predict the wide array of cognitive and psychosocial outcomes that would be highly relevant in the geriatric mental health context. Next, such data could be used to study how certain lifestyle and psychosocial factors moderate age-related changes in the brain. Additionally, neuroimaging data could also be used as an external dataset to validate hypotheses and prediction models derived from other datasets. As for associations between linguistic measures and cognitive functioning, previous studies had shown that patients diagnosed with semantic dementia utilize more abstract nouns and verbs as well as slower speech rates [12,13]. This highlights the possibility that such deterioration in language might also be present at the mild cognitive impairment (MCI) stage, which occurs before the dementia stage. Studying these associations would then inform us about the aspects of language to target to slow down the onset of dementia. Additionally, the joint investigation of linguistics, neuroimaging and psychosocial measures in the context of age-related cognitive impairment are vital as such research is scarce in both local and global contexts. Given that these measures have been previously identified as risk factors for cognitive impairment, the LFC study looked to further supplement this area of literature [5], [6], [7], [8],[12], [13], [14], [15]. Lastly, the study also examined if participation in previous NPIs from the CHI study would prevent cognitive decline and improve psychosocial outcomes when assessed during the LFC study. This would be a valuable addition to the CHI study as long-term individual and overall effects of NPIs on cognitive decline and psychosocial outcomes can be analysed and reported, providing key insights into the longitudinal effects of NPIs.

The present study adopted a prospective cohort study design, which allows for the longitudinal investigation of relationships between cognitive function, psychosocial outcomes as well as other multidisciplinary outcomes that were measured in the CHI study, over the 3-year period. Notably, not all measures in the CHI study were collected in the LFC study. This is because the LFC study focused more on cognitive functioning and its associated variables rather than establishing comprehensive health profiles of its participants. Data collection for the LFC study has been completed and the data analyses are currently ongoing.

## Research hypotheses

We hypothesized that: (1) there would be some cognitive decline in participants in the CHI study cohort after 3 years. Specifically, (i) there would be a larger proportion of MCI and dementia cases in the CHI study cohort after 3 years and (ii) cognitive scores of participants in the LFC study would be worse than their respective scores in the CHI study after controlling for age. We also hypothesized that (2) there would be differences in cognitive scores, psychosocial outcomes and speech characteristics between groups of normal ageing, MCI and dementia, and (3) selected neuroimaging, psychosocial and speech markers would be associated with cognitive decline and other psychosocial outcomes. Specifically, we hypothesized that (i) slower speech rates and more abstract nouns and verbs would be related to greater cognitive decline, (ii) patterns of functional and structural connectomes would be associated with cognitive decline as well as depression and anxiety symptoms. Lastly, we hypothesized that 4) participants who had been involved in NPIs from the CHI study would be likely to retain or show improvement in cognitive function and psychosocial outcomes when assessed during the LFC study. In support of this hypothesis, Ng et al. conducted a 5-year follow up and found that participants who had previously participated in NPIs had cognitive improvements as compared to control participants who did not participate in NPIs [16]. As such, the effects from previous NPIs should be retained when examined in the LFC Study. This paper describes the LFC study design, setting and procedures.

## Methods details

### Ethical approval

All procedures stated in this study were conducted in accordance with the Helsinki declaration and Singapore Guidelines for Good Clinical Practice. The National University of Singapore Institutional Review Board (NUS IRB-Reference Code: NUS-IRB-2021-162) provided ethical approval.

### Study sample and participation criteria

This LFC study recruited participants who were aged 60 to 99 years and had previously completed their neuropsychological assessments in the CHI study during February 2018 and January 2020 (inclusive). Additionally, these participants had indicated their consent to be re-contacted for future related studies and agreed to donate their coded data to be used in future research in their signed CHI study consent forms. Consequently, 653 participants fit the above criteria and were earmarked as recruitment targets in the LFC study. GPower analysis had shown that to provide 80 % power and detect a small effect size (*f^2^* = 0.02) at *α* = 0.05 using linear multiple regression with three predictors, 550 participants were required. Given that the LFC study looked to recruit 653 participants, this would provide sufficient power for subsequent analyses, where multiple regression analyses would be most commonly utilized.

The exclusion criteria of the LFC study included participants who had (1) withdrawn, (2) were uncontactable, or (3) diagnosed with dementia based on the study's consensus meetings with two expert clinicians and a neuropsychologist (KEH, RM and JY). If any exclusion criteria were met, participants were not recruited. As visit 2 of the LFC study specifically, participants who (1) were found to have Dementia in the LFC study, (2) reported to have any current or previous psychiatric and neurological conditions, or (3) reported any MRI contraindications would be excluded. Similar to the CHI study, the main research site for the LFC study was held at HAAC. Participants who were enrolled into the LFC study were given the option of being contacted for future research interventions. Additionally, these participants could decide whether they allowed their coded data to be used for further research collaborations with other investigators.

## Study procedure

### Informed consent

The LFC research team attempted to contact all eligible 653 older adults. This was done via phone calls, where the study procedures and purposes were introduced to them. Interested and eligible individuals were invited to HAAC subsequently. During their first visit to the HAAC, their mental capacity was screened through the administration of the Mini-Mental State Examination (MMSE) as stipulated by NUS Institutional Review Board (IRB) to ensure that potential participants possessed the mental capacity to provide informed consent to participate in the study. For those with primary school education and below, they would be deemed as having cognitive capacity if their MMSE scores are 20 and above. For participants with secondary school education and above, their MMSE scores should be 22 and above.

**Fig. 1:**
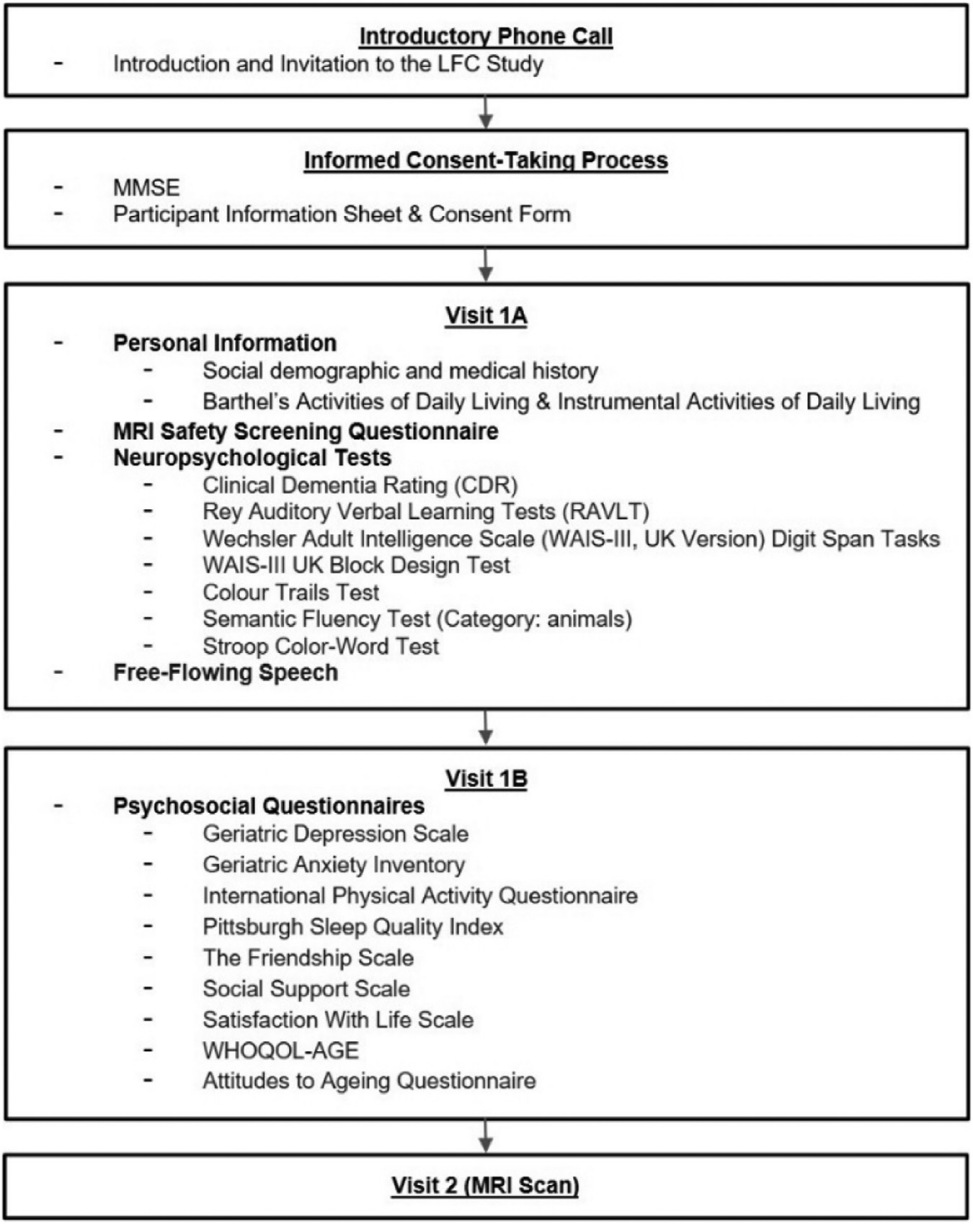
Summarized procedures and measures of the various visits in the LFC study.

Once the participants were deemed to possess the cognitive capacity to provide informed consent independently, a research team member explained the study details to them. A copy of the Participant Information Sheet (PIS) and Consent Form was also provided to participants. Participants were given ample time to consider before providing their written consent to participate in the study. Once their informed consent was taken, participants were invited to complete up to three separate visits, visits 1A, 1B and 2 (Fig. 1). These visits took an estimated total of 4 h to complete.

### Visit 1A

The first visit, visit 1A, was conducted at HAAC. Trained assessors, consisting of research assistants and a research nurse, recorded changes to the participant's socio-demographic and personal medical history information based on their CHI study records. Thereafter, participants underwent a safety screening questionnaire to gather information regarding their Magnetic Resonance Imaging (MRI) contraindications, such as metallic bodily implants, pacemakers and/or claustrophobia. The Barthel's Activities of Daily Living (BADL), instrumental Activities of Daily Living (iADL), the Clinical Dementia Rating (CDR) and neuropsychological tests were subsequently administered to the participants by trained assessors. A segment in the CDR required a face-to-face or phone interview with a family member or caregiver of the participants. As such, participants were requested to nominate a family member, who acted as an informant and provided objective information regarding the participant's cognition and daily functioning. The informant was first required to read and complete an Informant Information Sheet (IIS) and Consent Form before an interview was arranged with them. Lastly, participants who agreed to take part in a Free-Flowing Speech (FFS) segment were instructed to speak freely about topics of their choice, without any constraints for 15–20 min. They were allowed to speak using a language of their choice and their natural speech was audio recorded. Participants were also instructed to remain anonymous during the recording.

### Visit 1B

The second visit, visit 1B, consisted of psychosocial questionnaires and could be completed either at home or at HAAC. Participants were encouraged to complete visit 1B on paper or online (i.e., using Qualtrics) at home. Those who opted to complete the questionnaires on paper were given the questionnaires during visit 1A. Participants were also given the option to mail their completed questionnaires to a given address, or physically pass the questionnaires back to the research team. If participants were unable to complete the questionnaires at home, visit 1B could be conducted face-to-face at HAAC. Both visits 1A and 1B could be conducted in English or Mandarin, depending on the participant's preference and comfortability with language.

### Visit 2

The last visit, visit 2, was an MRI scan conducted at NUS Clinical Imaging Research Centre (CIRC). Due to budget constraints, this visit was made available to only 300 selected participants. Prior to the scan, participants were briefed on the scan procedures, as well as its potential risks and discomforts. The scan lasted for approximately 30 min. Once the scan concluded, participants were able to request for a copy of the MRI scan images before leaving the centre.

Participants were reimbursed after each visit. Reimbursement for visits 1A, 1B and 2 was $5, $5 and $10, respectively. Fig. 1 below summarizes the flow of the different visits, as well as the different instruments administered during each visit. The instruments utilized in this study were developed through the multi-disciplinary liaison of co-investigators, which allowed for the collection of multi-disciplinary measures.

Fig. 1 shows the flow and instruments administered during each study visit. The visit flow is divided into 5 parts: an introductory phone call, informed consent-taking process, visit 1A, 1B and 2. Participants were introduced and invited to HAAC during an introductory phone call. For the informed consent-taking process at HAAC, their cognitive capacity was screened via MMSE as per NUS-IRB requirements before the study was explained to them and their written informed consent were taken. Visits 1A, 1B and 2 were completed subsequently, consisting of neuropsychological assessments, psychosocial questionnaires and an MRI scan respectively.

### Consensus meeting for research diagnoses of cognitive status

Lastly, the cognitive statuses of participants were assessed via a two-step process. Preliminary research diagnoses were first obtained after the assessors administered the CDR and neurocognitive assessments in Visit 1A. Subsequently, final research diagnoses of normal ageing, MCI or dementia were made during the study's consensus meetings as stated earlier. The diagnoses of normal ageing were given for participants whose neurocognitive assessments scores were all above the z-score of −1.5. MCI and dementia diagnoses were based on Petersen's criteria for MCI and the Diagnostic and Statistical Manual of Mental Disorder (DSM-V) for dementia respectively [17]. Should adverse incidental findings arise from assessments, such as neuropsychological tests, depression/anxiety screening and the MRI scan, referral letters were then be provided to participants for further follow-up with clinicians. If the incidental finding from the MRI scans was deemed to be urgent by the CIRC team, participants were provided with a referral to the Emergency Room at National University Hospital. The data of participants with incidental findings may or may not be included in data analyses; this will be reviewed on a case-by-case basis.

### Outcome measures

The outcome measures as detailed below were collected in the LFC study. These measures are categorized into two types of variables: demographic and behavioural. For demographic variables, the participants’ age, sex, marital status, religion, education, employment status, financial status, personal medical history and functional status were recorded (Table 1). As for the psychosocial outcomes of behavioural variables, the depression and anxiety symptoms, sleep quality, life satisfaction, quality of life, attitudes towards ageing, social connectedness and perceived social support were measured (Table 2). In the cognitive domains, neuropsychological tests measuring working memory, cognitive and functional performance, short-term memory, selective visual attention, visual-spatial abilities, verbal fluency, selective attention as well as cognitive inhibition were administered. We also assessed participants physical activity via a questionnaire (Table 2). Finally, we collected audio recordings of the participants’ free-flowing speech (Table 2).

**Table 1:** Basic Demographic variables collected in the LFC Study.

Outcome Variable	Instrument	Visit
Age	Age was recorded based on the birth date shown on the National Registration Identification Card (NRIC).	1A
Sex	The participant's biological sex was recorded as male or female as stated on the participant's NRIC.	1A
Marital Status	Marital status was recorded as single, married, widowed or divorced/separated.	1A
Religion	Religion was categorized as Taoism/Buddhism, Christianity/Catholicism, Hinduism, Islam or others.	1A
Education	Education was measured by the years of formal schooling as well as highest education level, which was categorised as none, Primary, Secondary/Institute of Technical Education, Pre-University/Polytechnic or University.	1A
Employment Status	Employment status was self-reported by participants and categorised as retired, housewife, full-time, part-time or self-employed.	1A
Financial Status	Financial status was determined by the participant's housing type, current gross personal and household monthly income.	1A
Personal Medical History	In this section, any medical changes that had occurred since the participant's last visit were recorded. Participants were also asked to state the number of years that had occurred since the medical change.	1A
Functional Status	Barthel's Index of Activities of Daily Living (BADL) and Lawton's Instrumental Activities of Daily Living scale (iADL) were scales that measured older adults’ ability to carry out self-care tasks independently [18,19]. Both scales were scored on a 3-point ordinal scale (independent, some help required or dependent).	1A

**Table 2:** Behavioural variables collected in the LFC Study.

Outcome Variable	Instrument	Visit
Depression and Anxiety symptoms	Depression and anxiety symptoms were measured using the 15-item Geriatric Depression Scale and the 20-item Geriatric Anxiety Inventory [20,21]. Both scales had been validated in the local context and shown good reliability, where *α* = 0.80 and 0.94 respectively [22,23]. Both inventories were scored on a 2-point ordinal scale (yes/no; agree/disagree).	1B
Sleep Quality	Sleep quality was recorded using a 19-item Pittsburgh Sleep Quality Index [24]. It had been validated in the Chinese population and shown good psychometric properties [25].	1B
Life Satisfaction	Life satisfaction was assessed by a 5-item Satisfaction With Life Scale. Responses were scored on a 7-point Likert response scale [26]. This scale was established to have good reliability (*α* = 0.75) and validity in a local population [27].	1B
Quality of Life (QoL)	Quality of life was measured by the 13-item World Health Organization Quality Of Life (WHOQOL-AGE) questionnaire and scored on a 5-point Likert scale [28]. This questionnaire was previously administered to the ageing population and displayed good psychometric properties in terms of reliability (*α* = 0.91) and validity [29].	1B
Attitudes to Ageing	The 24-item Attitudes to Ageing Questionnaire was used to measure older adults’ attitudes towards ageing using a 5-point Likert scale [30]. This questionnaire had been shown to be psychometrically robust for use with older adults [30].	1B
Social Connectedness	Social connectedness was assessed by the 6-item Friendship Scale. Responses were scored on a 5-point Likert scale, and the scale possessed good reliability (*α* = 0.83), concurrent and discriminant validity [31].	1B
Perceived Social Support	Perceived social support was determined by a self-developed scale consisting of a single question (‘How many close friends/ relatives do you have?’) as well as seven items on perceived social support. Responses were scored on a 5-point Likert scale.	1B
Cognitive Functioning	Measures of cognitive functioning were as follows: (1) A locally modified and validated 30-point MMSE with local education and ethnic norms to assess global cognitive function [32,33]; (2) the 5-point CDR to measure cognitive and functional performance from interviews with participants and informants [34]; (3) a validated neurocognitive battery that measured auditory-verbal learning and short-term memory (Rey Auditory Verbal Learning Test), ability to hold and manipulate information in working memory (Digit Span Forward and Backward Task), selective visual attention (Colour Trails Test), visual-spatial abilities (Wechsler's Block Design), verbal fluency (Semantic Verbal Fluency—Animals) and selective attention as well as cognitive inhibition (Stroop Test Color and Word Test - Victoria Modification). Participants were diagnosed as either cognitively normal, MCI or dementia by a panel of expert clinician and psychologist.	1A
Free-Flowing Speech	Participants were asked to speak freely about a topic of their choice for 15–20 min. They were allowed to use a language of their choice and their speech will be audio recorded.	1A
Physical Activity	Physical activity level was measured by the 4-item International Physical Activity (short form) questionnaire [35]. Participants were asked to state the number of days and time they spent engaging in vigorous and moderate activities as well as walking and sitting. The questionnaire was validated with adults aged 18 to 65 across 12 countries, with an acceptable test-retest reliability of 0.74 [35].	1B

### MRI acquisition

Participants were scanned using 3T Siemens Magnetom Prisma scanner equipped with a 32-channel head coil. T1-weighted images were acquired using a MPRAGE protocol (TE = 2.45 ms; TR = 2200 ms; FOV = 263 × 350 × 350 mm; voxel size = 1 mm isotropic). Resting-state fMRI scans were acquired using a multiband EPI sequence (TR = 719 ms; TE = 30 ms; 60 axial slices; FOV = 220 × 220; voxel size = 2.5 mm isotropic). During the resting-state fMRI scan, subjects were instructed to remain awake and lie still with their eyes open while looking at a fixation cross. Additionally, gradient echo field maps (TR = 615 ms; TE1 = 4.92 ms; TE2 = 7.38 ms) were acquired for correcting the rsfMRI distortions. Diffusion-weighted images were acquired using a multi-shell multiband protocol consisting of 13 b0 images, 5 directions at b = 500 s/mm^2^, 48 directions at b = 1000 s/mm^2^ and 60 directions at b = 1000 s/mm^2^ (TE = 71 ms; TR = 3400 ms; phase encoding = *A*>>*P*; FOV = 232 × 232; 127 axial slices; voxel size = 1 × 1 × 2 mm). An additional b0 vol were acquired in the reversed encoding direction (*P*>>*A*) for the purpose of distortion correction. T2-weighted FLAIR sequences were acquired using a SPACE sequence (TR = 7000 ms; TE = 393 ms; TI = 2100 ms; 192 axial slices; FOV = 256 × 256 mm; voxel size = 1 mm isotropic).

### Data management

To protect their confidentiality, participants’ data, which consisted of questionnaire and assessment responses, MRI data and audio recordings, were de-identified. These data were kept anonymous and coded with a subject identification number (SID) assigned to each participant. Only the principal investigator (RM) and study coordinator (LZH) have access to their identifiers, which were recorded on a separate document. The link between the participants’ SID number and identifiers were kept separately from the data by the Principal Investigator and study coordinator. Research data that were collected in hardcopy would be entered and stored on a standalone computer. These softcopy data would also be password protected. Hardcopy data would be stored under lock and key, accessible to the selected personnel only.

### Image preprocessing

The T1 structural images will be preprocessed with FreeSurfer 7.2.0 using the default recon-all options. Briefly, this processing included the removal of non-brain tissue using a hybrid watershed/surface deformation procedure [36], automated Talairach transformation, segmentation of the subcortical white matter and deep gray matter volumetric structures (including hippocampus, amygdala, caudate, putamen, ventricles) [37,38], intensity normalization [39], tessellation of the gray matter white matter boundary, automated topology correction [36,40], and surface deformation [41,42]. Once the cortical models are completed, they will be registered to a spherical atlas which is based on individual cortical folding patterns to match cortical geometry across subjects [43], and the cerebral cortex will be parcellated into units with respect to gyral and sulcal structure. This method used both intensity and continuity information from the entire three-dimensional MR volume in segmentation and deformation procedures to produce representations of cortical thickness, calculated as the closest distance from the gray/white boundary to the gray/CSF boundary at each vertex on the tessellated surface [42]. The preprocessed cortical thickness images will subsequently resampled onto the fsaverage6 space and smoothed using a 5 mm kernel.

Resting-state fMRI scans will be preprocessed using fMRIPrep 22.0.2 [44]. Functional data will be slice time corrected using 3dTshift from AFNI [45] and motion-corrected using MCFLIRT [46]. This process will be followed by co-registration to the corresponding T1w using boundary-based registration [47] with 9 degrees of freedom, using bbregister from freesurfer. Motion correcting transformations, BOLD-to-T1w transformation, and T1w-to-template (MNI) warp will be concatenated and applied in a single step using antsApplyTransforms employing Lanczos interpolation. Subsequently, these preprocessed volumes are denoised by regressing out 6 motion parameters, the average signal of white matter and cerebrospinal fluid masks, global signal, and their derivatives, as well as cosines covering slow time drift frequency band using the ‘nilearn’ library in Python. Then, these volumes will be subjected to a 0.1 Hz low-pass filter. Scrubbing will be carried out using the Power approach [48] to further remove the effects of excessive head motion. These volumes will then be smoothed using a 5 mm FWHM kernel and subjected to a 0.1 Hz low-pass filter. Finally, the brainnetome atlas [49] will be used to parcellate the whole brain into 246 anatomical regions corresponding to the nodes of the network. Participants with excessive head motion (root mean squared displacement >0.25) will excluded from the analyses.

The preprocessing of the diffusion images, tractography, and construction of the structural connectome will be carried out using MRtrix3 (version 3.0.3) [50]. Briefly, MP-PCA denoising [51] and removal of Gibbs ringing artifacts [52] will be carried out on the raw images. Subsequently, they are corrected for motion, eddy currents and susceptibility-induced distortion using the inhomogeneity field maps obtained previously. Following this, bias field correction [53] will be carried out. Next, the gray matter (GM), white matter (WM), and cerebrospinal fluid (CSF) response functions will be obtained using the Dhollander algorithm [54] which are in turn used for estimating fiber orientation distributions (FOD) in the WM, GM and CSF tissues from diffusion data using spherical deconvolution. Then, anatomically-constrained tractography [55] will be carried out using the WM FOD. This involved the prior preparation of a GM mask from the Freesurfer segmentations and then using this mask to seed streamlines. The AAL-90 atlas [56] will be used to parcellate the whole brain into 90 regions for the construction of the structural connectome. The edges in the connectivity matrix will be operationalized as the number of streamlines connecting between each pair of regions.

## Data analysis

All demographic variables would be presented as descriptive summaries, such as means, standard deviations, median, standard errors and percentages, depending on the parametric/ non-parametric nature of the data. Non-parametric measures were normalized before being subjected to analyses.

To examine hypothesis 1, chi-square analyses were performed to investigate any changes in frequency of groups, i.e., normal ageing, MCI and dementia, across the 3-year period. Cognitive scores of participants from the LFC study were compared with their respective scores in the CHI study using dependent-samples *t*-tests.

As for hypothesis 2, potential differences of cognitive functioning, psychosocial outcomes and speech characteristics between groups, i.e., normal ageing, MCI and dementia, were analysed using independent-sample *t*-tests and Analysis of Variance (ANOVA).

To investigate hypothesis 3, cross-sectional brain-behaviour associations were examined via whole-brain approaches, n across various MRI modalities such as cortical thickness, structural connectivity resting state functional connectivity. Network-based statistics were used to identify structural and functional brain networks corresponding to behavioural and diagnostic variables. As for cortical thickness, whole-brain vertex-wise associations with behaviour will be analysed using the ‘standard linear regression’ module in the ‘BrainStat’ Python library [57]. The random field theory (RFT) correction, which corrects the probability of ever reporting a false positive result, was applied with a cluster-defining threshold of *p*<0.05.

Relationships between cognitive decline and various psychosocial outcomes such as depression and anxiety symptoms, quality of life and social connectedness were analysed using multiple regressions and structural equation models. Speech characteristics such as speech rate and concreteness were obtained after audio recordings are transcribed. Speech rates of various nouns and verbs were calculated by dividing the total number of nouns and verbs by the total number of minutes. Concreteness scores were obtained based on the concreteness ratings of 40,000 English words assembled by Brysbaert et al. [58]. Associations between these speech characteristics, cognitive decline and psychosocial outcomes were subsequently analysed using multiple regression analyses.

Lastly, to evaluate hypothesis 4, linear-mixed modelling and multivariate regression analyses were employed to examine the individual and overall effects of NPIs on cognitive functioning and psychosocial outcomes. P-values of <0.05 were considered statistically significant. Bonferroni corrections were conducted when applicable to reduce false positive rates.

Given that the LFC study has only recently completed data collection, data analyses are still ongoing and specific results are unavailable at the moment. Additionally, given that this is a protocol paper, the authors will focus more on the protocol rather than the results and findings.

## Discussion

The LFC study aimed to better understand the ageing process and its associated health issues by examining longitudinal changes in participants’ cognitive functioning and psychosocial outcomes as compared to the baseline CHI study. The present study also hoped to improve the mental and physical well-being of the older population in Singapore by investigating the associations between cognitive decline, psychosocial outcomes, speech characteristics and neuroimaging data. Significantly, the joint collection and investigation of these variables was not typically conducted in other cohort studies, including the CHI study. As such, this provides invaluable information regarding speech characteristics and neuroimaging data across the whole spectrum of dementia, ie., normal ageing, MCI and dementia. Findings would then be used to devise new intervention strategies, such as linguistics therapy, benefitting the overall mental and cognitive health of the Singapore ageing population.

The LFC study also evaluated if the effects of NPIs on cognitive impairment and psychosocial outcomes were still present at follow-up. This constituted a more rigorous evaluation of NPIs, since it provided vital information as to whether the effects of NPIs were sustained after non-pharmacological RCTs, i.e. dietary counselling and face-to-face mindfulness, were concluded. Reviews of NPIs have called for lengthier follow-up assessments to evaluate if clinical outcomes were maintained from the initial non-pharmacological RCTs [59,60]. The LFC study did so accordingly, making this another key strength of the current study.

Additionally, the LFC study assessed cognitive functioning through a two-step process, where CDR and neurocognitive assessments were administered before research diagnoses of normal ageing, MCI or dementia were made during consensus meetings with expert clinicians. This had been previously identified as one of the best available ways to identify those with a target condition amongst a participant pool [61]. Consensus panel members were also able to incorporate variables such as age, educational level, depression and anxiety symptoms and functional data alongside cognitive functioning to aid with the research diagnosis. In the case of disagreements within the panel, panel members also had the opportunity to discuss diagnostic considerations until consensus was reached. Coupled with the multi-disciplinary orientation of the consensus panel, this represented a robust and comprehensive method to assess cognitive status [62].

However, the conduct of the LFC study had coincided with the occurrence of the COVID-19 pandemic, which reduced participant recruitment numbers. This was especially so given that older adults are widely recognised as the vulnerable population during this pandemic [63]. Nevertheless, the research team pivoted to minimize the impact on participant recruitment numbers. Some participants had expressed concerns with heading outdoors due to high infection numbers present in the local community. Consequently, the study team allowed participants who were uncomfortable to turn up at HAAC to come at a later date. Thus, participants came at a date when they were comfortable, or when the infection numbers are lower. This minimized the impact on recruitment numbers, enabling the LFC study to provide sufficient information regarding cognitive functioning and psychosocial outcomes despite the ongoing pandemic.

Notably, the COVID-19 pandemic had also been associated with many outcome measures that are examined in this study, such as depression and anxiety symptoms, sleep quality and social support [64], [65], [66]. Hence, the data collected from the outcome measures might be affected by the occurrence of the pandemic. Nevertheless, this might be less of a concern given that data collection began in 2021, which was more than one year after the beginning of the pandemic. Additionally, some literature had also suggested that older adults were less affected in terms of mental health as compared to their younger adult counterparts [67,68]. Therefore, the impact of the pandemic on the data collected in the present LFC Study should be minimal.

## Conclusions

Collectively, the investigation of the effects of NPIs, as well as associations between cognitive decline, psychosocial outcomes, neuroimaging and speech data would contribute greatly to relevant literature and assist with the understanding of the ageing process. The findings of this study would then guide the development of new interventions and RCTs catered to older adults. Therefore, the LFC study should assist in improving the well-being of the Singapore ageing population, as well as global ageing and dementia science.

### Study progress

The LFC study has since completed data collection and data analyses are still ongoing. Given that this study has only recently completed data collection, no papers from this study have been published yet. This paper has its distinct scientific contribution in detailing the step-by-step procedures of the study. As the one of the first studies to jointly investigate neuroimaging, linguistics and psychosocial measures in the context of age-related cognitive impairment, this study could play a vital role in informing the development of interventions to delay cognitive decline in the local and global context.

## CRediT authorship contribution statement

**Zhi Hao Lim:** Writing – original draft, Writing – review & editing, Data curation, Project administration. **Ted Kheng Siang Ng:** Writing – review & editing, Supervision. **Zhiming Bao:** Resources, Writing – review & editing. **Junhong Yu:** Writing – review & editing, Resources. **Rathi Mahendran:** Conceptualization, Methodology.

## Declaration of competing interest

The authors declare that they have no known competing financial interests or personal relationships that could have appeared to influence the work reported in this paper.

## Data Availability

No data was used for the research described in the article.
